# Effects of land use, topography, climate and socio-economic factors on geographical variation pattern of inland surface water quality in China

**DOI:** 10.1371/journal.pone.0217840

**Published:** 2019-06-05

**Authors:** Qinghui You, Na Fang, Lingling Liu, Wenjing Yang, Li Zhang, Yeqiao Wang

**Affiliations:** 1 Key Laboratory of Poyang Lake Wetland and Watershed Research (Jiangxi Normal University), Ministry of Education, Nanchang, Jiangxi, China; 2 College of Life Sciences, Jiangxi Normal University, Nanchang, Jiangxi, China; 3 College of Geography and Environment, Jiangxi Normal University, Nanchang, Jiangxi, China; 4 Jiangxi Provincial Key Laboratory of Poyang Lake Comprehensive Management and Resource Development, Jiangxi Normal University, Nanchang, Jiangxi, China; 5 Department of Natural Resources Science, University of Rhode Island, Kingston, RI, United States of America; Imperial College London, UNITED KINGDOM

## Abstract

The deterioration of water quality has become a primary environmental concern worldwide. Understanding the status of water quality and identifying the influencing factors are important for water resources management. However, reported analyses have mostly been conducted in small and focused areas. It is still unclear if factors driving spatial variation in water quality would be different in extended spatial scales. In this paper, we analyzed spatial pattern of inland surface water quality in China using a dataset with four water quality parameters (i.e., pH, DO, NH_4_^+^-N and COD_Mn_) and the water quality level. We tested the effects of anthropogenic (i.e., land use and socio-economic) and natural (i.e., climatic and topographic) factors on spatial variation in water quality. The study concluded that the overall inland surface water quality in China was at level III (fair). Water quality level was strongly correlated with COD_Mn_ and NH_4_^+^-N concentration. In contrast to reported studies that suggested land use patterns were the determinants of inland surface water quality, this study revealed that both anthropogenic and natural factors played important roles in explaining spatial variation of inland surface water quality in China. Among the tested explanatory variables, mean elevation within watershed appeared as the best predictor for pH, while annual precipitation and mean air temperature were the most important explanatory variables for COD_Mn_ and DO, respectively. NH_4_^+^-N concentration and water quality level were most strongly correlated with the percent of forest cover in watershed. Compared to studies at smaller spatial scales, this study found different influencing factors of surface water quality, suggesting that factors may play different roles at different spatial scales of consideration. Therefore management policies and measures in water quality control must be established and implemented accordingly. Since currently adopted parameters for monitoring of inland surface water quality in China are largely influenced by natural variables, additional physicochemical and biological indicators are needed for a robust assessment of human impacts on water quality.

## Introduction

Inland surface water areas include different forms of open water bodies such as rivers and streams, lakes and reservoirs, permanent and seasonal wetlands. The deterioration of surface water quality has become a primary environmental concern worldwide, following the increasing demand of high-quality freshwater [1]. Inland surface water quality is considered to be influenced by a wide range of anthropogenic and natural factors, such as land use, social-economic status, topographic and climate variations [2, 3]. Understanding the status of surface water quality and identifying the key influencing factors are important for establishing policies for sustainable water resource management.

A number of studies have investigated relationships between water quality and landscape patterns. Human-influenced land uses have been considered as important drivers of water quality deterioration [4, 5]. Pollutant concentrations in water bodies have been reported to be positively correlated with agriculture and urban land use, but negatively related to cover types of natural vegetation [6–8]. The effects of land use types on water quality are varied in different geographical regions [2, 9]. For example, agricultural land use is considered as the primary factor for stream water nitrogen concentration [8], whereas other studies indicate that urban land use had the greatest influence on nitrogen and phosphorus in surface water bodies [10, 11].

Recent studies on ecology have analyzed the spatial structure of landscapes and the relationship of their configuration with water quality [12]. Reported studies have indicated that landscape configurations have significant influence on water quality of adjacent aquatic systems. For example, Lee et al. [13] indicated that water quality was likely to be degraded when there was high interspersion of various land use types existed within a watershed. Liu et al. [14] suggested that for a given total area, large and clustered agricultural or urban patches in the watershed would have greater impact on lake-water quality than small and scattered distributions.

Socio-economic factors, such as human population and gross domestic product (GDP), are often used to measure the intensity of human disturbances, so as to water quality parameters. Chen and Lu [15] considered that human population density and GDP explained 45% of spatial variation in water quality of a river in East China. The study also claimed that human population density was a fundamental predictor of phosphorus concentration. Morrice et al. [6] indicated that human population represented a major stress on coastal wetlands of Great Lakes and had a strong predictive ability for total phosphorus and chloride concentrations.

The impacts of natural environment on water quality, e.g., climate and topography, have been less studied in comparison with land use and socio-economic factors. Fukushima et al. [16] found that chemical oxygen demand in water of Lake Kasumigaura, the second largest lake in Japan, increased with rising air temperature. In addition, they observed that higher precipitation led to high nitrogen concentration, probably induced by both the surface runoff having high nitrogen concentration and the lowering of residence times of lake water. Topography has also been considered an influencing factor of inland surface water quality [17, 18]. For example, Pratt et al. [3] found that streams with a lower mean elevation generally had higher nitrate-nitrogen concentration, while mean slope exhibited a positive correlation with total solids.

However, fewer studies have tested the relative importance of above-mentioned multiple factors to address variation pattern of surface water quality in China. As one of the most water-rich countries in the world, China’s water resources are unevenly distributed geographically. Nationwide survey and evaluation of water quality started in the early 1980s. Monitoring of surface water quality has been implemented as a routine practice. Monitoring data derived from sampling sites have been published on weekly basis since 2006 by the government.

This study is aimed to identify the important factors influencing inland surface water quality in China. It is also to test if the driving factors of water quality would be different between smaller areas and extended spatial scales in China. Based on the results from reported studies, we hypothesize that (*H*_1_) areas with higher percent of human-influenced land uses have worse water quality; (*H*_2_) landscape diversity and fragmentation, and aggregation of human-influenced land uses have negative effects on water quality; (*H*_3_) high density of human population and GDP are associated with declining water quality; (*H*_4_) high precipitation increases nitrogen concentration in surface water, while the increase of air temperature lead to the increase in chemical oxygen demand; (*H*_5_) areas of high elevation and with gentle slopes have better water quality.

## Materials and methods

### Water quality dataset

Data contained four water parameters, i.e., pH, dissolved oxygen (DO), ammonium nitrogen (NH_4_^+^-N) and chemical oxygen demand in manganese (COD_Mn_), were obtained from the Ministry of Ecology and Environment of the People's Republic of China (http://123.127.175.45:8082/). These parameters are basic to life within aquatic ecosystems and relatively easy to measure using standard protocols, and were thus chosen as the indicators for weekly inland surface water quality monitoring across China. Water samples were collected by automatic samplers at each monitoring site and analyzed immediately following the protocols recommended in the Standard Methods for the Analysis of Water and Wastewater [19]. Water quality is classified into five levels, as summarized in Table 1, according to classification standards of surface water quality of China [20]. Water quality level is determined according to the principle of maximum membership grade, i.e., the category of the most impaired assessment factor is used as the comprehensive water quality classification. If there are assessment factors that cannot be assigned to any of the five water quality categories, the comprehensive water quality is considered as worse than level V (very poor).

**Table 1:** Criteria for the classification of inland surface water quality in China.

Water quality parameters	Level I (Excellent)	Level II (Good)	Level III (Fair)	Level IV (Poor)	Level V (Very poor)
pH	≥ 6.00 & ≤ 9.00	≥ 6.00 & ≤ 9.00	≥ 6.00 & ≤ 9.00	≥ 6.00 & ≤ 9.00	≥ 6.00 & ≤ 9.00
DO (mg/L)	≥ 7.50	≥ 6.00	≥ 5.00	≥ 3.00	≥ 2.00
NH_4_^+^-N (mg/L)	≤ 0.15	≤ 0.50	≤ 1.00	≤ 1.50	≤ 2.00
COD_Mn_ (mg/L)	≤ 2.00	≤ 4.00	≤ 6.00	≤ 10.00	≤ 15.00

**Fig 1:**
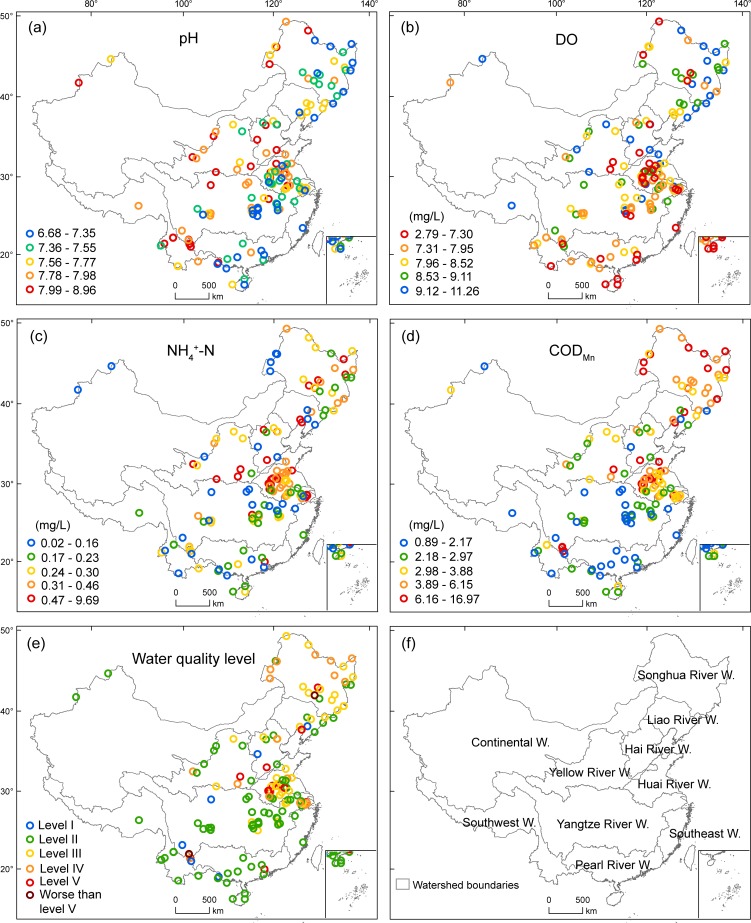
Values of four water quality parameters (a–d) and water quality level (e) at 145 monitoring sites in China, averaged from the weekly published data over the period between January 2014 and December 2016. (a–d) are in quantile classification. W. in (f) is the abbreviation for watershed. Maps are in Albers projection. Insets in the bottom right of maps show the south boundary of China, including all islands in the South China Sea.

Monitoring sites are located in the major lakes and rivers, and unevenly distributed to represent the inland surface water quality of ten major watersheds over the country (Fig 1; S1 Table). More monitoring sites are allocated in eastern China (e.g., the Huai River watershed) where human population density is high. The number of monitoring sites has increased from 82 in 2006 to 145 in 2016 (S1 Fig). In this study, water quality data from the 145 monitoring sites collected between January 2014 and December 2016 were used to ensure that data from different sites were temporally comparable. Weekly reported data were averaged to represent the overall water quality at each site in the study period. Water quality levels were recorded with the numbers from one to five in statistical analyses. If water quality was worse than level V, it was recorded with the number six. The overall water quality level at one site was determined by the mean value of weekly water quality levels (MWQL), i.e., MWQL < 1.50 as level I, 1.50 ≥ MWQL < 2.50 as level II, 2.50 ≥ MWQL < 3.50 as level III, 3.50 ≥ MWQL < 4.50 as level IV, 4.50 ≥ MWQL < 5.50 as level V, and MWQL ≥ 5.50 as worse than level V. Some monitoring sites had no data in dry seasons. The number of observations at each site varied between 48 and 160, with an average of 144 per site.

### Explanatory variables

The watershed boundary of each monitoring site was delineated from a 30 arc-second resolution digital elevation model (DEM, https://www.usgs.gov/centers/eros/science/usgs-eros-archive-digital-elevation-global-30-arc-second-elevation-gtopo30?qt-science_center_objects=0#qt-science_center_objects) using hydrology analysis tools in ArcGIS 10.2. Land use map was obtained from the Data Center for Resources and Environmental Sciences, Chinese Academy of Sciences (RESDC, http://www.resdc.cn/data.aspx?DATAID=184) at a spatial resolution of 1 km derived from the interpretation of Landsat 8 images acquired in 2015. The maximum likelihood (MLC) and support vector machine (SVM) classifiers were used for the initial land cover classification. The MLC assumes that a hyper-ellipsoid decision volume can be used to approximate the shape of the data clusters, while the SVM classifier is the most widely used non-parametric statistical learning classifier with no assumptions made regarding the underlying data distribution [21]. Land use types were divided into six categories of: (1) farmland, including dry land and paddy field; (2) forest; (3) grassland; (4) water, including rivers, lakes, reservoirs and ponds; (5) built-up land, including urban and industrial areas, rural settlements and roads; (6) unused land, including desert, marshland and bare soil. Percentage compositions of land use in the watershed of each monitoring site were extracted using zonal functions in ArcGIS 10.2. The percentages of grassland, water and unused land were small with mean values of 6%, 5% and 2%, respectively, and showed weak correlations with water quality parameters (Pearson’s |*r*| ≤ 0.12, *p* > 0.05). Therefore those land use categories were not considered in the subsequent statistical analyses.

Three landscape metrics, i.e., Shannon’s diversity index (SHDI), patch density and aggregation index (AI), were considered effective in measuring impacts on water quality [12–14]. SHDI is a measure of diversity in biological community, and indicates the patch diversity in landscape:

$$SHDI=\sum_{i=1}^{m}\left(p_{i}lnp_{i}\right)$$

where *p*_*i*_ is the proportion of the landscape occupied by land use type *i*, and *m* is the number of land use types in the landscape. Patch density is the number of patches per 100 hectare, and indicates landscape fragmentation. Aggregation index measures the tendency of a particular land use type to be aggregated:

$$AI=\left[\frac{g_{ii}}{\max-g_{\mathrm{ii}}}\times 100\%\right]$$

where *g*_*ii*_ is the number of like adjacencies (joins) between pixels of land use type *i* based on the single-count method, and max-*g*_*ii*_ is the maximum number of like adjacencies (joins) between pixels of land use type *i* based on the single-count method [22]. Those landscape metrics were calculated using land use data with the function “ClassStat” in R package “SDMTools” (https://cran.r-project.org/web/packages/SDMTools/index.html).

Data of human population density and GDP for the year 2015 at 1 km spatial resolution were obtained from RESDC (http://www.resdc.cn/data.aspx?DATAID=251, http://www.resdc.cn/DOI/doi.aspx?DOIid=33). Climate variables including annual precipitation and mean air temperature for the year 2015 were also obtained from RESDC at a spatial resolution of 30 arc-seconds (http://www.resdc.cn/data.aspx?DATAID=229, http://www.resdc.cn/data.aspx?DATAID=228). Mean elevation and slope within the watershed of each monitoring site were calculated from the DEM model with the aid of surface tool in ArcGIS 10.2.

### Statistic analyses

First, the normality of all variables was examined using normal probability plots and Kolmogorov-Smirnov tests. NH_4_^+^-N, COD_Mn_, water quality level, percent of built-up land, human population density, GDP and slope were log-transformed to achieve normality because they had highly skewed distributions. Pairwise relationships between explanatory variables and relationships between water quality (pH, DO, NH_4_^+^-N, COD_Mn_ and water quality level) and each explanatory variable were examined using Pearson’s correlation analysis.

Ordinary least squares regressions (OLS) were then applied to explore the overall explanatory power of the environmental variables on each water quality parameter and water quality level. The Akaike information criterion (AIC) [23] and step-wise backward selection were used to identify the most parsimonious multi-predictor models. Moran’s *I* correlograms and global Moran’s *I* values were used to evaluate the pattern and strength of spatial autocorrelation in model residuals [24]. Significant spatial autocorrelation was found among the residuals of OLS models (S2 Fig). Spatial autocorrelation might inflate type I error (false negative) rates and bias parameter estimates. Therefore, spatial simultaneous autoregressive (SAR) models were employed to examine the spatial autocorrelation in the data with the function “errorsarlm” in R package “spdep” (https://cran.r-project.org/web/packages/spdep/index.html). SAR models of the error type were chosen with a lag distance of 300 km and weighted neighborhood structure. The selection of lag distance was based on the trade-off between AIC values and the number of monitoring sites having no neighbors within the distance class [25]. Pseudo-*R*^2^ (hereafter *R*^2^) values for SAR models were calculated as the squared Pearson correlation between predicted and observed values [26].

The relative importance of each predictor in the most parsimonious model, which assesses the explanatory power of the predictor while controlling for the effects of other predictors, was investigated using the function “calc.relimp” with metric “pmvd” in the R package “relaimpo” [27]. The metric “pmvd” calculates a weighted average of sequential *R*^2^ values over all possible models (i.e., all combinations of predictors in the model). The *R*^2^ value of each model was partitioned into relative proportions explained by each predictor. The relative proportions were then multiplied by the *R*^2^ of the model to obtain the absolute fraction of *R*^2^ value explained by a particular variable. To account for spatial autocorrelation, a standard SAR model was first performed. The spatial component of the fitted values was then removed, and fitted values excluding the spatial component were entered as a new response variable in the *R*^2^ partitioning procedure [28].

## Results

### Geographical patterns of water quality

Water quality parameters and level varied considerably over geographical space and watersheds (Fig 1; S1 Table). The pH values were between 6.67 and 8.96 (mean = 7.68), all in a normal range for inland surface water (Fig 1A). DO values varied from 2.79 mg/L to 11.26 mg/L, with a mean of 8.26 mg/L (Fig 1B). About 93% of the sites had DO ≥ 6.00 mg/L, which is one of threshold values for level II water quality (Table 1). NH_4_^+^-N concentrations ranged between 0.02 mg/L and 9.69 mg/L, with an average of 0.52 mg/L (Fig 1C). COD_Mn_ values varied from 0.89 mg/L to 16.97 mg/L (mean = 4.14 mg/L), and varied considerably among watersheds (Fig 1D; S3 Fig; S1 Table). High COD_Mn_ values were mostly concentrated in eastern and northeastern China, i.e., Songhua River and Huai River watersheds, both the major industrial and agricultural regions of China.

About 4% of the monitoring sites (*n* = 6) were classified with level I water quality, 48% with level II (*n* = 70), 24% with level III (*n* = 34), 15% with level IV (*n* = 21), and 8% with level V (*n* = 12) (Fig 1E). About 1% of the monitoring sites (*n* = 2) had water quality worse than level V. The overall water quality in China was rated as Level III (MWQL = 2.80). Water quality level varied largely among watersheds (Fig 1E; S3 Fig; S1 Table). Poor water quality (levels IV, V and worse than level V) appeared mostly in Huai River and Songhua River watersheds, whereas good water quality (levels I and II) was more common in the watersheds of southern China. All water quality parameters except pH were significantly correlated with each other and water quality level (Table 2). COD_Mn_ and NH_4_^+^-N had relatively high correlations with water quality level (Pearson’s *r* = 0.87 and 0.65, respectively, *p* < 0.001), suggesting that water quality level was largely determined by COD_Mn_ and NH_4_^+^-N.

**Table 2:** Pairwise Pearson’s correlation coefficients for four water quality parameters and water quality level.

	pH	DO	NH_4_^+^-N	COD_Mn_	Water quality level
pH	1	-	-	-	-
DO	0.25**	1	-	-	-
NH_4_^+^-N	-0.03	-0.37***	1	-	-
COD_Mn_	0.15	-0.18*	0.54***	1	-
Water quality level	0.08	-0.38***	0.65***	0.87***	1

*, *p* < 0.05

**, *p* < 0.01

***, *p* < 0.001.

### Relationships between water quality and explanatory variables

Pearson’s correlation analyses showed that among the tested explanatory variables, pH had the strongest and positive correlation with elevation (*r* = 0.45, *p* < 0.001; Table 3). pH was also significantly correlated with temperature and precipitation, but did not show significant correlations with land use and social-economic variables. DO was most strongly correlated with temperature (*r* = -0.33, *p* < 0.001), whereas its correlations with other explanatory variables were relatively low. COD_Mn_ had the strongest correlation with precipitation (*r* = -0.46, *p* < 0.001), and had slightly lower correlations with temperature, SHDI and patch density (*r* ranging between -0.42 and -0.44, *p* < 0.001).

**Table 3:** Pearson’s correlations between water quality parameters, water quality level and explanatory variables.

Explanatory variables	pH	DO	NH_4_^+^-N	COD_Mn_	Water quality level
Farmland	-0.06	-0.07	0.40***	0.27**	0.26**
Forest	-0.16	0.04	-0.40***	-0.33***	-0.41***
Built-up land	-0.11	-0.17*	0.38***	0.14	0.21*
SHDI	0.03	0.16	-0.31***	-0.42***	-0.34***
Patch density	-0.12	-0.15	-0.14	-0.44***	-0.32***
AI (farmland)	-0.12	-0.12	0.03	0.25**	0.19*
AI (built-up land)	-0.07	-0.08	0.04	0.15	0.11
GDP	-0.13	-0.28***	0.33***	0.01	0.14
Human population density	-0.10	-0.25**	0.32***	-0.05	0.15
Temperature	-0.26**	-0.33***	-0.11	-0.44***	-0.25**
Precipitation	-0.33***	-0.27**	-0.16	-0.46***	-0.26**
Elevation	0.45***	0.20*	-0.20*	-0.02	-0.21*
Slope	0.12	0.10	-0.39***	-0.36***	-0.39***

SHDI, Shannon’s diversity index; AI (farmland), aggregation index for farmland; AI (built-up land), aggregation index for built-up land; GDP, gross domestic product.

*, *p* < 0.05

**, *p* < 0.01

***, *p* < 0.001.

In contrast, NH_4_^+^-N had the strongest relationships with land use composition variables (Table 3). The effect of forest on NH_4_^+^-N was negative (*r* = -0.40, *p* < 0.001), while that of farmland and built-up land was positive (*r* = 0.40 and 0.38, respectively, *p* < 0.001). NH_4_^+^-N also showed significant correlations with socio-economic and topographic variables. Water quality level was significantly correlated with land use, climate and topographic variables.

The multivariate SAR models explained 19%– 45% of the spatial variation in water quality parameters and water quality level (Table 4). The highest *R*^2^ appeared to the model for COD_Mn_, whereas the lowest *R*^2^ occurred in the model for DO. Elevation was the most important variable in the model for pH, while temperature and precipitation were the strongest predictors in the models for DO and COD_Mn_, respectively. Forest was the most important variable in the models for NH_4_^+^-N and water quality level.

**Table 4:** Multi-predictor spatial simultaneous autoregressive (SAR) models for four water quality parameters and water quality level.

	Estimate	SE	*z*	*P*-value	*R*^2^	Partitioned *R*^2^
**pH**					0.32	
Temperature	3.42e^-02^	8.87e^-03^	4.64	< 0.001		0.07
Precipitation	-6.99e^-04^	1.30e^-04^	-5.17	< 0.001		0.09
Elevation	2.57e^-04^	4.59e^-05^	5.54	< 0.001		0.12
Spatial signals	-	-	-	-		0.04
**DO**					0.19	
Built-up land	-0.64	0.15	-3.54	< 0.05		0.03
SHDI	0.87	0.28	3.03	< 0.05		0.03
GDP	-0.72	0.14	-4.11	< 0.01		0.05
Temperature	-3.52e^-02^	7.26e^-03^	-4.67	< 0.001		0.07
Spatial signals	-	-	-	-		0.01
**NH**_**4**_^**+**^**-N**					0.30	
Forest	-0.18	3.03e^-02^	-5.41	< 0.001		0.11
SHDI	-0.42	0.15	-3.61	< 0.05		0.04
Human population density	0.36	0.52e^-02^	4.81	< 0.001		0.08
Temperature	-4.71e^-02^	1.15e^-03^	-4.09	< 0.01		0.05
Spatial signals	-	-	-	-		0.02
**COD**_**Mn**_					0.45	
Forest	-8.22e^-02^	1.69e^-02^	-4.97	< 0.001		0.08
SHDI	-0.49	0.62e^-02^	-5.47	< 0.001		0.11
AI (farmland)	0.76	0.21	3.63	< 0.05		0.04
Precipitation	-5.57e^-04^	2.38e^-05^	-5.74	< 0.001		0.14
Slope	-5.77e^-02^	1.71e^-02^	-3.99	< 0.05		0.05
Spatial signals	-	-	-	-		0.03
**Water quality level**					0.43	
Forest	-0.57	4.83e^-02^	-5.58	< 0.001		0.12
SHDI	-0.23	6.91e^-02^	-4.28	< 0.01		0.06
Human population density	7.66e^-02^	2.57e^-02^	2.92	< 0.05		0.03
Precipitation	-3.93e^-04^	0.71e^-05^	-5.09	< 0.001		0.09
Elevation	-1.13e^-04^	4.34e^-05^	-2.90	< 0.05		0.03
Slope	-0.14	4.20e^-05^	-4.73	< 0.001		0.07
Spatial signals	-	-	-	-		0.03

SHDI, Shannon’s diversity index; GDP, gross domestic product; AI (farmland), aggregation index for farmland.

## Discussion

This study showed that variables of land use composition had significant relationships with NH_4_^+^-N, COD_Mn_ and water quality level, whereas their relationships with pH and DO were relatively weak (Tables 3 and 4). Forest was negatively correlated with NH_4_^+^-N, COD_Mn_ and water quality level, while farmland and built-up land were positively correlated with them. This observation is consistent with most of reported studies [7, 11] and thus supports *H*_1_. In contrast with previous studies indicating either farmland [6] or built-up land [15] as the primary land use predictor for surface water quality, this study found that forest had stronger explanatory power than farmland and built-up land, and was the most important predictor in the multivariate models for NH_4_^+^-N and water quality level (Table 4). This may be because higher percent of forest means less human-influenced land cover and therefore less potential sources of pollution. Moreover, forest helps maintain high water quality through minimizing soil erosion, thus reducing sediment in water bodies, and through trapping or filtering other water pollutants. In addition, densely growing plants in forest can absorb and concentrate pollutants (e.g., nitrogen and phosphorus) from water, while highly diversified microbial communities in surface litter, debris and organically enriched soil can degrade the pollutants efficiently.

SHDI and patch density were found to be negatively correlated with NH_4_^+^-N, COD_Mn_ and water quality level (Tables 3 and 4), which does not support the first part of *H*_2_ that landscape diversity and fragmentation have negative effects on water quality. The result may be explained by positive relationships between SHDI, patch density and forest (S2 Table), i.e., watersheds with more land use types and fragmented landscapes generally had higher percent of forest cover. Aggregation indices of farmland and built-up land were negatively correlated with water quality although the relationships were weak (Table 3), which is in line with previous findings that clustered agricultural or urban patches had larger influences on water quality than scattered ones [14], and thus support the other part of *H*_2_ that human-influenced land uses have negative effects on water quality.

Human population density and GDP showed positive correlations with NH_4_^+^-N and water quality level, and negative correlations with DO (Tables 3 and 4), thus supporting *H*_3_. In contrast, only weak correlations were found between social-economic variables and COD_Mn_ (Table 3). COD_Mn_ is an indicative measure of oxidizable organic matter in surface water. In addition to human-introduced pollutants (e.g., organic fertilizers and pesticides), organic matter produced by photosynthesis in biological organisms is an important type of pollutant in surface water [29]. The rate and efficiency of photosynthesis are influenced by a wide range of variables, including water transparency, temperature and concentration of carbon dioxide, which may not be strongly correlated with human population density and GDP.

Annual precipitation appeared as the best predictor for and was negatively correlated with COD_Mn_ (Tables 3 and 4), which is consistent with the findings in other studies [30, 31]. High precipitation including rainfall and snow melting may cause a “dilution effect” that lowers the concentrations of organic matter and other pollutants (e.g., nutrients) in surface water [30]. For example, a negative correlation was observed between precipitation and NH_4_^+^-N in our study (Table 3). Low nutrient concentrations will in turn limit the growth of biological organisms (e.g., phytoplankton) and consequently hamper the accumulation of organic matter in an aquatic ecosystem [32].

A negative correlation was also observed between COD_Mn_ and temperature (Table 3). Temperature and precipitation were highly correlated (S2 Table). After controlling for the effect of precipitation by partial correlation analysis [33], the relationship between temperature and COD_Mn_ became insignificant. This result is inconsistent with reported studies stating that temperature was an important factor influencing organic contamination in surface water [32, 34]. The results therefore do not support *H*_4_ that high precipitation increases nitrogen concentration in surface water, while the increase of air temperature leads to the increase in COD_Mn_.

This study found that elevation was the strongest explanatory variable for pH (Tables 3 and 4), i.e., surface water at high elevation generally had higher pH values (slightly alkaline). Monitoring sites at high elevation are usually located in mountainous areas where water flows down through streams and picks up alkaline minerals from rocks in the streams [35, 36]. As pollutants increase in downstream, microbial degradation of pollutants produces acidic compounds that lower water pH values [37]. In addition, elevation and slope were negatively correlated with NH_4_^+^-N, COD_Mn_ and water quality level (Tables 3 and 4), indicating that watersheds of high elevation and with steeper slopes usually had better water quality, which therefore only partly supports *H*_5_. This result is different from previous findings that mean slope within a watershed exhibited a negative relationship with water quality [3, 38]. It was explained that gentle slope acted as a literal sink for particulates and other pollutants by slowing or entirely stopping runoff. In this study, slope was strongly correlated with farmland and forest (S2 Table), i.e., watersheds with gentle slopes are usually plain areas with high percent of farmland, while those with steep slopes are mountainous areas well covered with forest. The land use and topographic patterns may explain the different relationship between slope and water quality in China.

The *R*^2^ of multivariate SAR models varied from 0.19 to 0.45, and the lowest *R*^2^ appeared to the model for DO (Table 4). Temperature was the strongest predictor for DO in the model (Tables 3 and 4), which is consistent with the recognition that the solubility of oxygen decreases as water temperature increases [39–41]. Other abiotic and biotic factors that may affect DO, such as water salinity, atmospheric pressure and photosynthesis in algae and aquatic plants [42–44], were not considered in the model. The relatively low *R*^2^ of multivariate models suggest that other factors such as point sources of pollution may be more important for explaining the spatial variation of inland surface water quality in China. However, it is difficult to obtain such data at a national scale.

The results show that different hypotheses are not mutually exclusive but many factors can act synergistically as drivers of spatial pattern variation of inland surface water quality in China. In contrast with reported studies indicating that land use patterns were the main factors influencing surface water quality [6, 7], especially those conducted in specific areas of China [12, 15], our study found that natural environment (i.e., climate and topography) were important in explaining spatial variation of the tested water quality parameters and water quality level in China. This may be because most of the reported studies were conducted in relatively small areas where climatic and topographic heterogeneity might not be the influencing factor, while the effects of land use and social-economic factors were thus more prominent. Our results suggest that human-related factors are important when developing policies for water resources management at local scales, while natural environmental factors should be considered for policy development at larger spatial scales. In addition, the effect of an explanatory variable on surface water quality may not be the same in different areas, which may be influenced by social, economic and cultural contexts, as well as climatic and topographic conditions of the study area. Our results also suggest that climate change may have profound impact on inland surface water quality. Assessment of the impacts of climate changes is important for developing adaptive management options [39, 45, 46].

## Conclusions

Analysis of field-based weekly published monitoring data showed that the overall inland surface water quality in China was rated as level III (fair). More monitoring sites in watersheds of southern China indicated level II (good), while multiple sites in Songhua River watershed in northeastern China and Huai River watershed between Yellow and Yangtze Rivers in eastern China showed the level IV (poor) and even worse level V (very poor). Water quality level was mainly determined by COD_Mn_ and NH_4_^+^-N, suggesting that organic matter and nitrogen were the major types of pollutants in China’s inland surface water. The main explanatory variables varied considerably for the tested water quality parameters and water quality level. Among the tested explanatory variables, elevation was the most important explanatory variable for pH, while air temperature and precipitation were the strongest predictors for DO and COD_Mn_, respectively. NH_4_^+^-N concentration and water quality level were most strongly correlated with the percent of forest cover in watersheds that was highly inversely proportional to the percent of human-influenced land cover. In general, mountainous forested areas with high precipitation showed better water quality than lowland areas with high percent of human-influenced landscape. Compared to studies carried out at local spatial scales, this study found different influencing factors of surface water quality, suggesting that factors may play different roles at different spatial scales. Therefore management policies and measures in water quality control must be established and implemented accordingly. Since currently adopted parameters for monitoring of inland surface water quality in China are influenced by climatic and topographic variables, additional physicochemical and biological indicators are needed for a robust assessment of human impacts on water quality. For example, total nitrogen and phosphorus, and the content of pathogenic bacteria in surface water are considered to be mainly influenced by agricultural and domestic pollution [7, 20], and can be included as additional indicators for surface water quality.

## Supporting information

S1 Fig Number of monitoring sites for inland surface water quality in China from 2006 to 2016. (DOCX)

S2 Fig Moran's *I* correlograms for four water quality parameters and water quality level. (DOCX)

S3 Fig COD_Mn_ and water quality level in ten major watersheds of China. (DOCX)

S1 Table Water quality parameters, water quality level and explanatory variables in ten major watersheds of China. (DOCX)

S2 Table Pairwise Pearson’s correlations between explanatory variables. (DOCX)
