# Supplementary figures and images for: Effects of land use, topography, climate and socio-economic factors on geographical variation pattern of inland surface water quality in China
Source: PLoS One. 2019 Jun 5;14(6):e0217840. doi: 10.1371/journal.pone.0217840 (PMC6550451; doi:10.1371/journal.pone.0217840)

**S1 Fig.** Number of monitoring sites for inland surface water quality in China from 2006 to 2016.


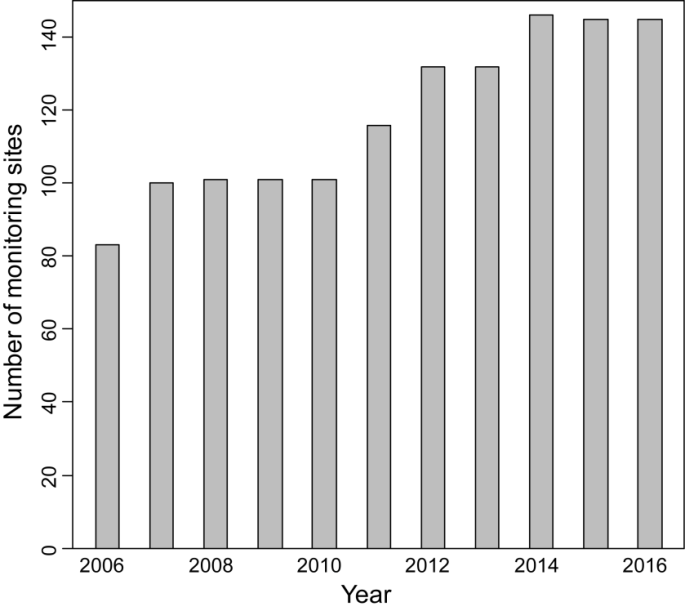

Supplement: S1 Fig — (DOCX) [file pone.0217840.s001.docx]
