# Supplementary material for: Effects of land use, topography, climate and socio-economic factors on geographical variation pattern of inland surface water quality in China
Source: PLoS One. 2019 Jun 5;14(6):e0217840. doi: 10.1371/journal.pone.0217840 (PMC6550451; doi:10.1371/journal.pone.0217840)

**S2 Fig.** Moran's *I* correlograms for four water quality parameters and water quality level (black), residuals from ordinary least-squares (OLS) models (red), and residuals from spatial simultaneous autoregressive (SAR) models (blue). Global Moran's *I* value (*I*_g_, calculated with a neighbor distance of 300 km) significance: ***, *p* < 0.001; **, *p* < 0.01; *, *p* < 0.05; ns, not significant.


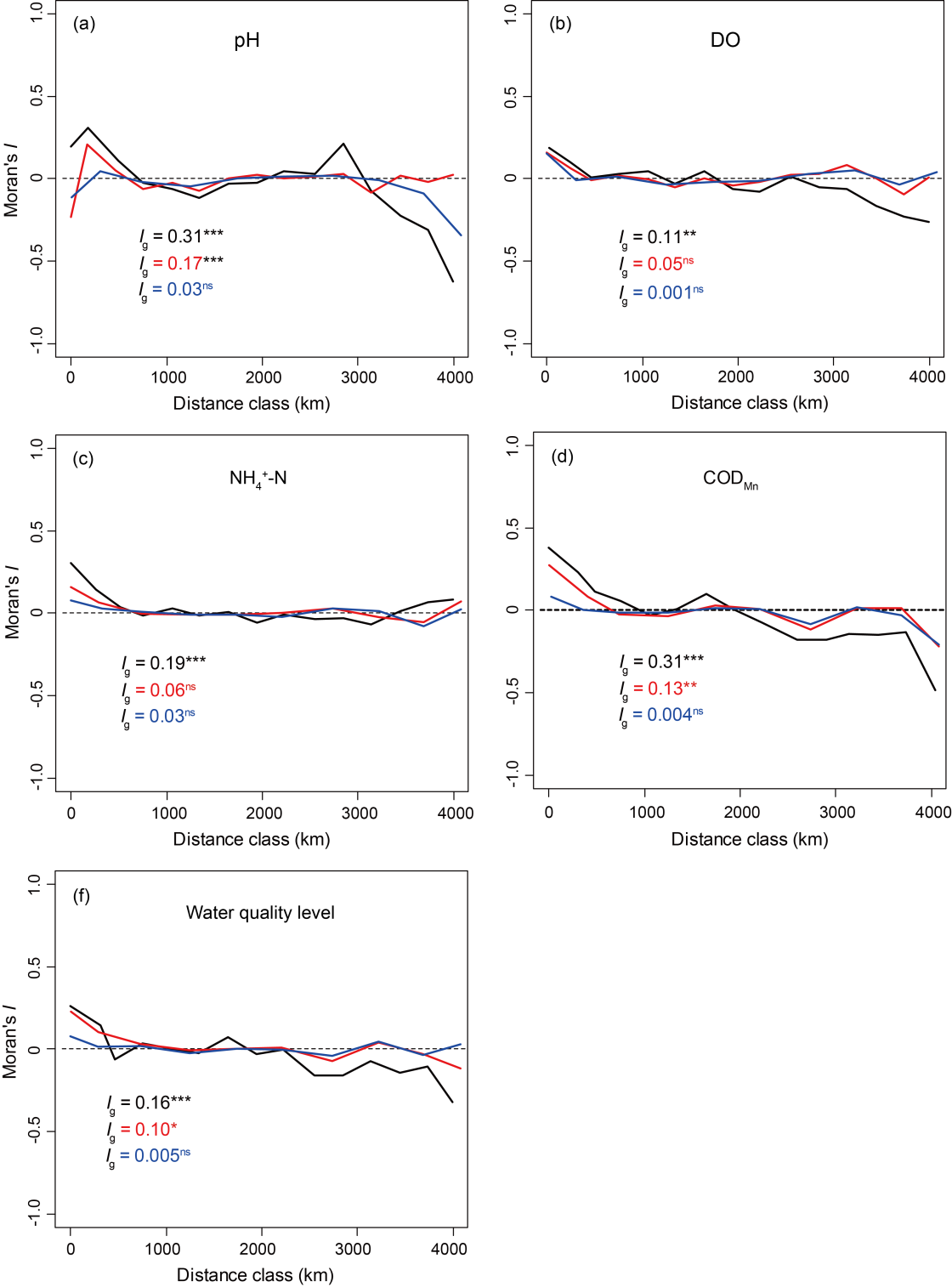

Supplement: S2 Fig — (DOCX) [file pone.0217840.s002.docx]
