# Supplementary material for: Effects of land use, topography, climate and socio-economic factors on geographical variation pattern of inland surface water quality in China
Source: PLoS One. 2019 Jun 5;14(6):e0217840. doi: 10.1371/journal.pone.0217840 (PMC6550451; doi:10.1371/journal.pone.0217840)

**S3 Fig**. COD_Mn_ (a) and water quality level (b) in ten major watersheds of China. Watersheds are sorted in a descending order by the latitudes of their geographical centroids. A, Songhua River watershed; B, Liao River watershed; C, Continental watershed; D, Hai River watershed; E, Yellow River watershed; F, Huai River watershed; G, Yangtze River watershed; H, Southeast watershed; I, Southwest watershed; J, Pearl River watershed. Range bars show maximum and minimum of non-outliers; boxes are inter-quartile ranges (25% ile to 75% ile); bold bars are medians; circles are outliers.


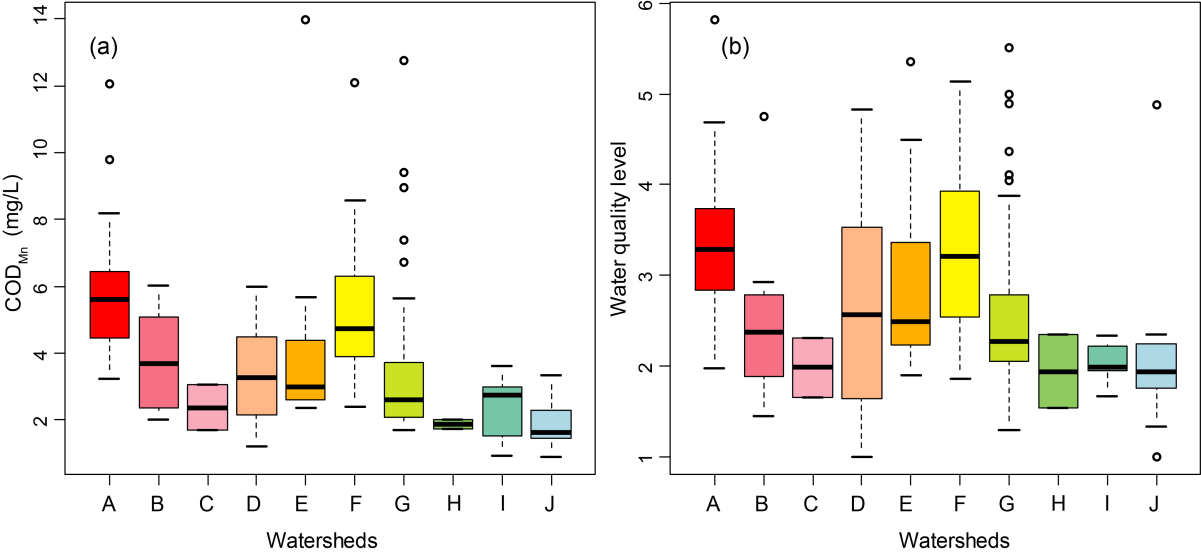

Supplement: S3 Fig — (DOCX) [file pone.0217840.s003.docx]
