# Supplementary material for: Effects of land use, topography, climate and socio-economic factors on geographical variation pattern of inland surface water quality in China
Source: PLoS One. 2019 Jun 5;14(6):e0217840. doi: 10.1371/journal.pone.0217840 (PMC6550451; doi:10.1371/journal.pone.0217840)
Supplement: S1 Table — (DOCX) [file pone.0217840.s004.docx]

**S1 Table** Water quality parameters, water quality level and explanatory variables in ten major watersheds of China.

| Explanatory variables | Yangtze River W. | Hai River W. | Huai River W. | Yellow River W. | Liao River W. | Continental W. | Songhua River W. | Southwest W. | Southeast W. | Pearl River W. |
| --- | --- | --- | --- | --- | --- | --- | --- | --- | --- | --- |
| pH | 7.69 | 7.79 | 7.74 | 8.05 | 7.52 | 7.95 | 7.55 | 7.85 | 7.11 | 7.32 |
| DO (mg/L) | 8.04 | 9.06 | 8.49 | 8.79 | 9.59 | 9.69 | 8.41 | 7.95 | 7.98 | 7.30 |
| COD (mg/L) | 2.40 | 2.93 | 4.95 | 4.22 | 3.75 | 2.26 | 5.81 | 1.96 | 1.89 | 1.86 |
| NH_4_^+^-N (mg/L) | 0.25 | 0.37 | 0.51 | 0.92 | 0.48 | 0.13 | 0.63 | 0.20 | 0.14 | 0.24 |
| Water quality level | 2.14 | 2.29 | 3.10 | 2.84 | 2.55 | 1.82 | 3.40 | 2.03 | 1.94 | 2.16 |
| Percent of farmland | 0.43 | 0.31 | 0.66 | 0.37 | 0.45 | 0.12 | 0.37 | 0.13 | 0.16 | 0.28 |
| Percent of forest | 0.37 | 0.35 | 0.05 | 0.10 | 0.38 | 0.32 | 0.44 | 0.41 | 0.70 | 0.55 |
| Percent of built-up land | 0.04 | 0.03 | 0.15 | 0.07 | 0.06 | 0.00 | 0.03 | 0.01 | 0.03 | 0.06 |
| Shannon’s diversity index | 1.90 | 1.95 | 1.47 | 1.92 | 1.61 | 2.07 | 1.55 | 1.92 | 1.63 | 1.90 |
| Patch density | 0.16 | 0.15 | 0.12 | 0.15 | 0.12 | 0.10 | 0.08 | 0.10 | 0.20 | 0.16 |
| Aggregation index for farmland | 7.64 | 7.89 | 8.91 | 8.29 | 7.57 | 9.73 | 9.17 | 9.68 | 6.38 | 7.26 |
| Aggregation index for built-up land | 5.40 | 4.02 | 4.13 | 4.17 | 3.94 | 4.45 | 5.23 | 5.18 | 4.12 | 4.36 |
| GDP (Yuan/km^2^) | 702.11 | 543.63 | 1282.15 | 1198.68 | 967.64 | 61.06 | 308.40 | 77.85 | 876.45 | 1684.09 |
| Human population density (*n*/km^2^) | 392.49 | 170.81 | 588.59 | 291.71 | 257.98 | 61.09 | 123.20 | 61.87 | 276.74 | 488.94 |
| Annual mean temperature (℃) | 18.12 | 11.73 | 15.46 | 12.57 | 6.20 | 13.35 | 3.45 | 18.37 | 18.54 | 22.52 |
| Annual precipitation (mm) | 1088.19 | 572.00 | 744.75 | 441.09 | 694.71 | 779.50 | 532.39 | 987.00 | 1470.50 | 1471.90 |
| Elevation (m) | 208.24 | 208.63 | 135.50 | 803.00 | 183.25 | 838.00 | 196.17 | 1062.80 | 56.00 | 67.73 |
| slope | 3.50 | 3.93 | 0.77 | 2.01 | 1.73 | 6.43 | 1.55 | 7.48 | 4.96 | 2.46 |

W. is the abbreviation for watershed.
