# Supplementary material for: Effects of land use, topography, climate and socio-economic factors on geographical variation pattern of inland surface water quality in China
Source: PLoS One. 2019 Jun 5;14(6):e0217840. doi: 10.1371/journal.pone.0217840 (PMC6550451; doi:10.1371/journal.pone.0217840)
Supplement: S2 Table — (DOCX) [file pone.0217840.s005.docx]

**S2 Table** Pairwise Pearson’s correlations between explanatory variables for inland surface water quality in China.

|  | Farmland | Forest | Built-up land | SHDI | PD | AI | AI | GDP | POP | Temperature | Precipitation | Elevation | RANGE | Slope |
| --- | --- | --- | --- | --- | --- | --- | --- | --- | --- | --- | --- | --- | --- | --- |
|  |  |  |  |  |  | (farmland) | (built-up land) |  |  |  |  |  |  |  |
| Farmland | 1.00 | - | - | - | - | - | - | - | - | - | - | - | - | - |
| Forest | -0.67 | 1.00 | - | - | - | - | - | - | - | - | - | - | - | - |
| Built-up land | 0.81 | -0.64 | 1.00 | - | - | - | - | - | - | - | - | - | - | - |
| SHDI | -0.46 | 0.19 | -0.29 | 1.00 | - | - | - | - | - | - | - | - | - | - |
| Patch density | -0.08 | 0.18 | 0.20 | 0.45 | 1.00 | - | - | - | - | - | - | - | - | - |
| AI(farmland) | -0.01 | -0.14 | -0.27 | 0.01 | -0.58 | 1.00 | - | - | - | - | - | - | - | - |
| AI(built-up land) | -0.18 | -0.10 | -0.17 | 0.05 | -0.32 | 0.62 | 1.00 | - | - | - | - | - | - | - |
| GDP | 0.64 | -0.45 | 0.80 | -0.13 | 0.40 | -0.44 | -0.27 | 1.00 | - | - | - | - | - | - |
| POP | 0.63 | -0.43 | 0.78 | -0.14 | 0.42 | -0.42 | -0.22 | 0.82 | 1.00 | - | - | - | - | - |
| Temperature | 0.11 | 0.08 | 0.29 | 0.18 | 0.59 | -0.45 | -0.25 | 0.46 | 0.57 | 1.00 | - | - | - | - |
| Precipitation | -0.03 | 0.31 | 0.10 | 0.17 | 0.53 | -0.50 | -0.42 | 0.30 | 0.41 | 0.84 | 1.00 | - | - | - |
| Elevation | -0.49 | 0.22 | -0.65 | 0.18 | -0.24 | 0.42 | 0.12 | -0.63 | -0.60 | -0.40 | -0.41 | 1.00 | - | - |
| RANGE | -0.51 | 0.47 | -0.57 | 0.42 | 0.09 | 0.14 | 0.07 | -0.42 | -0.32 | 0.08 | 0.08 | 0.61 | 1.00 | - |
| Slope | -0.76 | 0.72 | -0.63 | 0.53 | 0.19 | -0.02 | 0.05 | -0.54 | -0.50 | 0.01 | 0.12 | 0.55 | 0.78 | 1.00 |

SHDI, Shannon’s diversity index; AI (farmland), aggregation index for farmland; AI (built-up land), aggregation index for built-up land; GDP, gross domestic product; POP, human population density. Colors show the significance of *r* values. Green, *p* < 0.05; orange, *p* < 0.01; red, *p* < 0.001; blue, insignificant.
